# Clinical characteristics and mortality risk factors of mixed bacterial infections in hematopoietic stem cell transplantation recipients

**DOI:** 10.3389/fcimb.2023.1223824

**Published:** 2023-09-18

**Authors:** Yanfeng Liu, Yi Liu, Xuefeng Chen, Yan Jia

**Affiliations:** ^1^ Department of Hematology, Xiangya Hospital, Central South University, Changsha, China; ^2^ Department of Clinical Pharmacology, Xiangya Hospital, Central South University, Changsha, China; ^3^ National Clinical Research Center for Geriatric Disorders, Xiangya Hospital, Central South University, Changsha, China; ^4^ Department of Hematology, The Third Xiangya Hospital of Central South University, Changsha, China

**Keywords:** mixed bacterial infections, hematopoietic stem cell transplantation, drug resistance, mortality, risk factors

## Abstract

**Background and objective:**

Mixed bacterial infections (MBI) is one of the complications after hematopoietic stem cell transplantation (HSCT) and increases the risk of patient death. However, there are few reports specifically on this topic. The purpose of this study was to investigate the clinical characteristics and mortality risk factors of MBI in HSCT recipients.

**Methods:**

The electronic medical records of patients undergoing HSCT were collected. The epidemiological features and antibiotic resistance of patients with and without MBI were compared. Logistic regression and Cox regression were used to identify the risk factors for MBI acquisition and death. R language was used to construct a prediction model for the overall survival of HSCT recipients with MBI.

**Results:**

The cumulative incidence of MBI was 6.3% and the mortality was 48.8%. Time interval from diagnosis to transplantation > 180 days (HR=2.059, 95% CI 1.042-4.069, *P*=0.038) and ICU admission after transplantation (HR=2.271, 95% CI 1.053-4.898, *P*=0.036) were independent risk factors for MBI acquisition. Engraftment period > 20 days (HR=2.273, 95% CI 1.028-5.027, *P*=0.043), continuous renal replacement therapy (HR=5.755, 95% CI 1.691-19.589, *P*=0.005) and septic shock (HR=4.308, 95% CI 2.085-8.901, *P*=0.000) were independent risk factors associated with mortality.

**Conclusions:**

MBI has become a serious problem that cannot be ignored after HSCT. It is urgent for clinicians to pay high attention to it and formulate reasonable monitoring and treatment plans to improve the prognosis of patients.

## 1 Introduction

Hematopoietic stem cell transplantation (HSCT) is one of the greatest advances in modern medicine, which can help patients recover normal hematopoietic and immune function. Therefore, it is an important treatment for hematological malignancies, solid tumors, genetic metabolic diseases, primary immunodeficiency diseases, etc.(Bazinet and Popradi, 2019; Carreras et al., 2019; Castagnoli et al., 2019). Although in the past few decades, with the advent of a series of new drugs and the progress of transplantation technology, the success rate of HSCT has been significantly improved. However, infection remains one of the most common complications and early causes of death after transplantation (Esquirol et al., 2021).

Due to the use of high-dose radiotherapy, chemotherapy and immunosuppressive agents, almost all transplant patients will suffer from agranulocytosis, impaired mucosal barrier and weakened immune function. In addition, factors such as central venous catheter implantation, parenteral nutrition and long-term hospitalization greatly increase the chance of bacterial invasion (Ferreira et al., 2018; Cao et al., 2021). In a review of literature, we found that the distribution pattern of bacterial infections in HSCT recipients reported in different countries varied widely: the ratio of gram-positive bacteria (GPB) to gram-negative bacteria (GNB) ranged from 85% *vs.*15% to 26% *vs.*74% (Balletto and Mikulska, 2015). Even in the same country, the data reported by different transplant centers are quite different. For example, in China, Cao WJ et al. found that GNB accounted for an absolute dominant position (71.67%) in bloodstream infections (BSI) in HSCT recipients (Cao et al., 2021). However, the statistical results of Wang D et al. showed that the proportion of GPB and GNB infections was close (49.06% *vs.* 50.94%) (Wang et al., 2019). Although some epidemiological and clinical data related to bacterial infections in HSCT populations have been previously published, most of them were concentrated in a single category (such as carbapenem-resistant *Klebsiella pneumoniae* and methicillin-resistant *Staphylococcus aureus*), few studies specifically focused on mixed bacterial infections (MBI), which hinders the effective management and prognosis improvement of transplant patients.

In this study, we analyzed the distribution, antibiotic resistance and prognosis of MBI in HSCT recipients in the hematology ward of a large general hospital in China over the past 10 years, and paid special attention to the risk factors of MBI acquisition and death, which provided valuable information for a comprehensively understanding of the characteristics of post-transplant infection and adjusting anti-infection treatment strategies.

## 2 Materials and methods

### 2.1 Study design and data collection

The clinical information of patients who underwent HSCT with MBI in the Department of Hematology, Xiangya Hospital from January 1, 2012 to September 30, 2021 was collected through the electronic medical record system. The follow-up time was 100 days after infection.

This was a retrospective study. The Institutional Review Committee of Xiangya Hospital endorsed this project and approved the patient’s waiver of informed consent (No. 2019030162). Researchers kept patient information strictly confidential.

### 2.2 Definitions

Bacterial infection was defined as the isolation of microorganisms from blood or other sterile body fluids (e.g. bronchoalveolar lavage fluid, urine, cerebrospinal fluid, etc.), accompanied by clinical evidence of infection and excluding other possible causes (Horan et al., 2008; Patriarca et al., 2017). MBI referred to the simultaneous or sequential (within 72 hours) isolation of two or more different bacterial strains from a patient. If the isolation interval of multiple strains exceeded 72 hours, it was identified as multiple distinct episodes of bacterial infection (Trifilio et al., 2015). For common skin contaminants (Coagulase-negative *Staphylococci* (CoNS), *Propionibacterium acnes*, *Micrococcus species*, and *Corynebacterium species* except *C. jeikeium*), or isolation of *Bacillus species* and non-hemolytic *Streptococci*, repeat culture and identification were required within 72 hours (Gjaerde et al., 2017). Carbapenem resistance was defined as the minimum inhibitory concentration (MIC) of meropenem and/or imipenem ≥ 4 mg/mL (Durante-Mangoni et al., 2019). Multidrug resistance (MDR) was defined as insensitivity to three or more antimicrobial agents (excluding natural resistance) (Bentivegna et al., 2021). Inappropriate empirical antimicrobial therapy was defined as the absence of microbial-sensitive antibiotics within 48 hours of specimen extraction (Jia et al., 2023). Sepsis shock was defined as persistent hypotension on the basis of severe sepsis, despite sufficient volume replacement (Forcina et al., 2017). Neutrophil engraftment was defined as neutrophil > 0.5×10^9^/L for at least 3 consecutive days. Platelet engraftment was defined as platelet > 20×10^9^/L for at least 7 consecutive days without blood transfusion (Huo et al., 2020).

### 2.3 Infection prevention and treatment strategies

Patients were admitted to the laminar air-flow wards after a medicated bath, received a daily sterile diet and oral, eye, nasal, and perianal care. Except for 3 patients admitted before 2015, all patients were routinely screened for carbapenem-resistant Enterobacteriaceae by rectal swabs on admission. Five days before transplantation, oral administration of nystatin 50 wu and gentamicin 8 wu (three times a day) for intestinal preparation.

During transplantation, if the patient was in agranulocytosis status or was expected to develop agranulocytosis within 48 hours, but had no obvious symptoms of infection such as fever, cough and diarrhea, meropenem or imipenem would be given for bacterial infections prevention. If the patient had infection-related clinical manifestations, carbapenems could be used alone or in combination with tigacycline, vancomycin, linezolid, etc. In the late stage of transplantation, with the engraftment of donor stem cells and the relief of infection symptoms, antibiotics were de-escalated to β-lactamase inhibitor compound preparation, cephalosporins or quinolones (Jia et al., 2023).

### 2.4 Graft versus host disease prevention strategies

All patients were treated with an anti-rejection regimen based on antithymocyte globulin (ATG) + cyclosporin A (CsA) + mycophenolate mofetil (MMF) + short-course methotrexate (MTX): The total amount of ATG was 7.5 mg/kg, which was used three days before stem cell infusion. CsA and MMF were given at the beginning of pretreatment. The former was started at 5 mg/kg/d, and the dose was dynamically adjusted according to the blood concentration to maintain a level of 180-200 ng/mL (if no obvious GVHD, the dose was reduced after 3 months and discontinued after 6 months). The initial dose of the latter was 1g/d, halved at +30 d (the day of stem cell infusion was considered day 0) and stopped at +45 d to +60 d. MTX was administered at 15 mg/m^2^ (+ 1 d) and 10 mg/m^2^ (+ 3 d, + 6 d, + 11 d).

### 2.5 Microbiological analysis

The collection of specimens was strictly in accordance with aseptic operation principles. Bacterial strains were identified by VITEK 2 compact automatic microbiological analyzer, and the antimicrobial susceptibility was determined by MIC method and Kirby Bauer diffusion method. The test results were interpreted according to the standards published by the American Committee for Clinical and Laboratory Standardization (CLSI) (Cao et al., 2021).

### 2.6 Construction and validation of prognosis models

The independent risk factors related to death were used as clinical variables, and the 100 days overall survival (OS) was used as clinical outcome endpoint. A nomogram model was constructed by Cox regression analysis and R language. The consistency index (C-index) was used to compare the similarity between the predicted value and the observed value. The calibration plot was applied to assess the sensitivity and specificity of the prediction model (Jia et al., 2023).

### 2.7 Statistical analysis

All statistical analysis was conducted by IBM SPSS 24.0 software. The continuous variables were compared by independent sample *t* test and Mann-Whitney U test, the categorical variables were compared by chi-square test or Fischer’s exact test. The “rms” software package of R language was used to construct the nomogram. The receiver operating characteristic (ROC) curve was plotted and the area under the curve (AUC) was calculated to evaluate the prediction accuracy of this model. Kaplan-Meier analysis and Log-rank test were used to compare the difference of survival curve between patients with and without risk factors. *P* < 0.05 was considered statistically significant.

## 3 Results

### 3.1 Patient characteristics

From January 1, 2012 to September 30, 2021, a total of 1269 cases of HSCT were performed in our hospital, of which 80 were included in this study. The cumulative incidence of MBI was 6.3%, including 45 males (56.2%) and 35 females (43.8%), with an average age of 34.5 years. *K. pneumoniae* + *Acinetobacter baumannii* and *Staphylococcus* + *Escherichia coli* were the most common combination of mixed infections. About 56.2% of infections occurred during agranulocytosis. Forty-five patients (56.2%) received inappropriate empirical anti-infective therapy, and 39 patients (48.8%) died within 100 days after infection.

Compared with single bacterial infection, patients with MBI had longer platelet engraftment time (22 d *vs.* 18 d, *P* = 0.004), higher creatinine level (63.95 mmol/L *vs.* 57.75 mmol/L, *P* = 0.049), higher incidence of intensive care unit (ICU) admission (28.7% *vs.* 11.8%, *P* = 0.002) and septic shock (31.2% *vs.* 14.0%, *P* = 0.002), and worse outcomes (mortality 48.8% *vs.* 27.9%, *P* = 0.002) (**Table 1**). At the same time, we also compared MBI with multiple distinct episodes of bacterial infection, and the results showed that there was no significant difference in the above indicators between the two groups (*P* > 0.05). Moreover, to understand the impact of different infection sites on patient clinical outcomes, we compared the 100-day mortality of MBI patients originating from bloodstream and lungs. Our data showed that the former was significantly higher than the latter (59% *vs.* 41%, *P* = 0.044).

**Table 1:** Comparison of clinical characteristics between HSCT recipients with and without MBI.

Characteristic	Non-mixed bacterial infections (n=136)	Mixed bacterial infections (n=80)	*P*
Sex, n (%)			0.565
Female	65 (47.8%)	35 (43.8%)	
Male	71 (52.2%)	45 (56.2%)	
Age, years, median (IQR)	27 (18, 39.25)	34.5 (19, 46.25)	0.054
Primary disease, n (%)			0.097
Acute lymphocytic leukemia	30 (22.1%)	19 (23.8%)	
Acute myelogenous leukemia	32 (23.5%)	29 (36.2%)	
Myelodysplastic syndrome	14 (10.3%)	6 (7.5%)	
Aplastic anemia	26 (19.1%)	11 (13.8%)	
Lymphoma	3 (2.2%)	3 (3.7%)	
Thalassemia	13 (9.6%)	6 (7.5%)	
Others	18 (13.2%)	6 (7.5%)	
Infection sites, n (%)			0.139
Lung	46 (33.8%)	25 (31.2%)	
Bloodstream	78 (57.4%)	53 (66.2%)	
Urine	8 (5.9%)	0 (0%)	
Others	4 (2.9%)	2 (2.5%)	
Human leukocyte antigen-identical, n (%)	51 (37.5%)	31 (38.8%)	0.855
Multidrug resistance, n (%)	99 (72.8%)	62 (77.5%)	0.443
Acute graft-versus-host disease, n (%)	63 (46.3%)	34 (42.5%)	0.585
Infection during agranulocytosis, n (%)	68 (50%)	45 (56.2%)	0.374
Inappropriate empiric antimicrobial treatment, n (%)	70 (51.5%)	45 (56.2%)	0.497
Time of neutrophil engraftment, median (IQR)	13 (11.00, 16.00)	13.5 (12.00, 16.00)	0.564
Time of platelet engraftment, median (IQR)	18 (13.00, 23.25)	22 (14.75, 28.00)	0.004*
Indicators within 24 h of infection			
Neutrophil count, 10^9^/L, median (IQR)	0.45 (0.00, 2.90)	0.05 (0.00, 3.20)	0.391
Lymphocyte count, 10^9^/L, median (IQR)	0.1 (0.00, 0.60)	0 (0, 0.525)	0.201
Platelet count, 10^9^/L, median (IQR)	27 (14.75, 57.75)	27 (16.75, 60.50)	0.765
Procalcitonin, mg/L, median (IQR)	0.395 (0.15, 1.71)	0.654 (0.21, 2.51)	0.192
Albumin, g/L, mean ± SD	34.17 ± 5.78	32.94 ± 5.19	0.117
Total bilirubin, mmol/L, median (IQR)	12.15 (7.28, 18.53)	11.25 (8.28, 20.15)	0.923
Creatinine, mmol/L, median (IQR)	57.75 (46.98, 73.13)	63.95 (52.88, 76.00)	0.049*
Hospital stay, days, median (IQR)	53 (37.00, 74.25)	48.5 (34.75, 79.25)	0.911
Intensive care unit admission, n (%)	16 (11.8%)	23 (28.7%)	0.002*
Septic shock, n (%)	19 (14.0%)	25 (31.2%)	0.002*
Mortality, n (%)	38 (27.9%)	39 (48.8%)	0.002*

IQR, interquartile range; *P values are statistically significant.

### 3.2 Overview of pathogens

**Figure 1:**
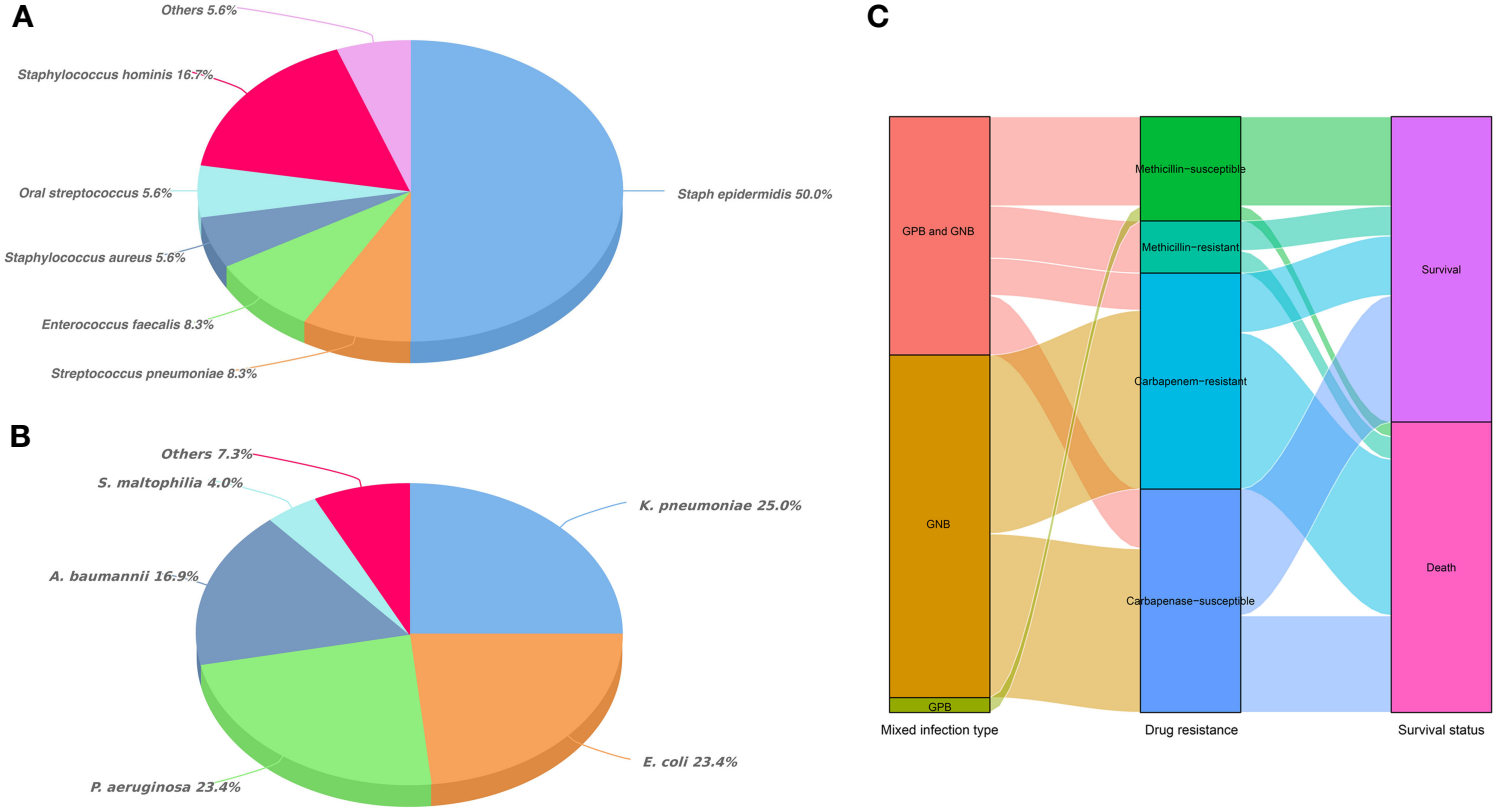
Distribution of mixed bacterial infections in HSCT recipients. **(A)** Classification and percentage of Gram-positive bacteria in mixed bacterial infections; **(B)** Classification and percentage of Gram-negative bacteria in mixed bacterial infections; **(C)** Trajectory tracking Sankey diagram among infection type, antibiotic resistance and survival.

Overall, *Staphylococcus* accounted for an absolute advantage (72.3%) in GPB of MBI (**Figure 1A**). Among the GNB of MBI, *K. pneumoniae*, *E. coli*, *Pseudomonas aeruginosa* and *Acinetobacter baumannii* accounted for 25.0%, 23.4%, 23.4% and 16.9%, respectively (**Figure 1B**). In terms of antibiotic sensitivity, GPB was still sensitive to vancomycin and linezolid, and only one streptococcus strain was resistant to vancomycin. However, the situation of GNB was not so optimistic. Data showed that *E. coli*, *K. pneumoniae* and *A. baumannii* were generally resistant to amoxicillin, gentamicin, ciprofloxacin, levofloxacin, sulfamethoxazole, tobramycin, ceftriaxone and other commonly used antibiotics. The resistance rate of *A. baumannii* to meropenem and imipenem was even more than 85%. Although tigecycline and amikacin remain sensitive to most GNB, they were “powerless” to *P. aeruginosa* and *Stenotrophomonas maltophilia*, respectively (**Tables 2**, **3**). **Figure 1C** showed the trajectory tracking among mixed infection type, antibiotic resistance and survival.

**Table 2:** Antibiotic resistance of gram-positive bacteria in MBI.

Antimicrobial	Staphylococcus (n=26)	Streptococcus (n=5)	Enterococcus (n=3)	Others (n=2)
Penicillins, %	34.6	60	33.3	66.6
Gentamicin, %	23.1	–	50	0
Erythromycin, %	76.9	80	100	66.7
Levofloxacin, %	61.5	40	80	0
Ciprofloxacin, %	61.5	–	66.7	0
Vancomycin, %	0	20	0	0
Nitrofurantoin, %	0	0	33.3	–
Sulfamethoxazole, %	65.4	80	–	66.7
Linezolid, %	0	0	–	–

“–” means no relevant data.

**Table 3:** Antibiotic resistance of gram-negative bacteria in MBI.

Antimicrobial	*E. coli* (N=29)	*A. baumannii* (N=21)	*K. pneumoniae* (N=31)	*P. aeruginosa* (N=29)	*S. maltophilia* (N=5)	Other (N=9)
Amoxicillin, %	65.5	100.0	67.7	100.0	100.0	77.8
Amikacin, %	6.9	47.6	9.7	0.0	100.0	22.2
Ciprofloxacin, %	82.8	85.7	67.7	13.8	–	44.4
Gentamicin, %	72.4	76.2	58.1	6.9	100.0	55.6
Levofloxacin, %	86.2	81.0	61.3	17.2	20.0	44.4
Sulfamethoxazole, %	75.9	66.7	77.4	100.0	20.0	66.7
Tobramycin, %	72.4	66.7	64.5	0.0	–	55.6
Piperacillin-tazobactam, %	31.0	81.0	25.8	31.0	100.0	100.0
Cefoperazone-sulbactam, %	31.0	66.7	29.0	41.4	60.0	22.2
Ceftriaxone, %	79.3	100.0	74.2	100.0	100.0	33.3
Tigecycline, %	0.0	14.3	19.4	100.0	–	11.1
Meropenem, %	20.6	95.2	25.8	58.6	100.0	11.1
Imipenem, %	24.1	85.7	16.1	62	100.0	22.2

“–” means no relevant data.

### 3.3 Risk factors for MBI acquisition

In univariate logistic regression analysis, age (*P* = 0.046), primary disease (*P* = 0.039), time interval from diagnosis to transplantation > 180 days (*P* = 0.006), time of platelet engraftment > 20 days (*P* = 0.009), urethral catheterization (*P* = 0.007), and ICU admission (*P* = 0.002) were associated with the acquisition of MBI. In multivariate logistic regression analysis, only the time interval from diagnosis to transplantation > 180 days (HR = 2.059, 95% CI 1.042-4.069, *P* = 0.038) and ICU admission (HR = 2.271, 95% CI 1.053-4.898, *P* = 0.036) were identified as independent risk factors for MBI acquisition after HSCT (**Table 4**).

**Table 4:** Univariate and multivariate logistic analysis of MBI acquisition.

Variable	Univariate analysis		Multivariate analysis	
	OR (95% CI)	*P*	OR (95% CI)	*P*
Sex	1.177 (0.676,2.051)	0.565		
Age	1.02 (1,1.04)	0.046*	1.019 (0.998,1.04)	0.083
Primary disease	0.903 (0.819,0.995)	0.039*	0.903 (0.812,1.004)	0.059
History of relapse/refractory state	1.862 (0.856,4.047)	0.117		
Time interval from diagnosis to transplantation > 180 days	2.411 (1.289,4.509)	0.006*	2.059 (1.042,4.069)	0.038*
Neutrophil count < 0.5×10^9^/L 1 month before transplantation	1.235 (0.557,2.74)	0.603		
Human leukocyte antigen-identical	1.054 (0.597,1.861)	0.855		
Time of platelet engraftment > 20 days	2.119 (1.208,3.715)	0.009*	1.734 (0.935,3.215)	0.081
Time of neutrophil engraftment > 14 days	1.061 (0.61,1.843)	0.835		
Urethral catheterization	2.655 (1.31,5.382)	0.007*	1.676 (0.761,3.69)	0.200
Acute graft-versus-host disease	0.856 (0.491,1.495)	0.585		
Mesenchymal stem cell infusion	1.228 (0.605,2.493)	0.569		
Continuous renal replacement therapy	1.737 (0.422,7.145)	0.444		
Intensive care unit admission	3.026 (1.485,6.166)	0.002*	2.271 (1.053,4.898)	0.036*
Hospital stay > 60 days	1.089 (0.622,1.906)	0.767		

OR, odds ratio; CI, confidence interval. *P values are statistically significant.

### 3.4 Risk factors for MBI death

Of the 80 HSCT recipients with MBI, 39 died within 100 days after infection. The comparison between the death group and the survival group was shown in **Table 5**. Time interval from diagnosis to transplantation > 180 days (*P* = 0.018), engraftment period > 20 days (*P* = 0.001), continuous renal replacement therapy (CRRT) (*P* = 0.000), mechanical ventilation (*P* = 0.000) and septic shock (*P* = 0.000) were significantly different between the two groups in univariate analysis. In multivariate Cox analysis, engraftment period > 20 days (HR = 2.273, 95% CI 1.028-5.027, *P* = 0.043), CRRT (HR = 5.755, 95% CI 1.691-19.589, *P* = 0.005) and septic shock (HR = 4.308, 95% CI 2.085-8.901, *P* = 0.000) were 3 independent risk factors associated with mortality.

**Table 5:** Univariate and multivariate Cox analysis of risk factors for 100-day mortality of MBI.

Variable	Univariate analysis		Multivariate analysis	
	HR (95% CI)	*P*	HR (95% CI)	*P*
Sex	1.325 (0.694,2.528)	0.394		
Age	1.007 (0.986,1.028)	0.518		
Primary disease	1.085 (0.976,1.207)	0.131		
Types of infection	0.625 (0.321,1.216)	0.166		
Infection sites	0.96 (0.768,1.201)	0.723		
Multidrug resistant	0.842 (0.399,1.773)	0.650		
Inappropriate empiric antimicrobial treatment	1.058 (0.562,1.992)	0.862		
Time interval from diagnosis to transplantation > 180 days	3.476 (1.234,9.795)	0.018*	1.61 (0.512,5.067)	0.416
Human leukocyte antigen-identical	0.728 (0.374,1.418)	0.351		
Acute graft-versus-host disease	0.886 (0.47,1.671)	0.708		
Time of engraftment > 20 days	3.497 (1.654,7.391)	0.001*	2.273 (1.028,5.027)	0.043*
Indicators within 24 h of infection				
Neutrophil count < 0.5×10^9^/L	1.065 (0.565,2.006)	0.845		
Platelet count < 20×10^9^/L	1.45 (0.753,2.792)	0.266		
Procalcitonin > 5 μg/L	1.069 (0.507,2.253)	0.861		
Total bilirubin > 34.2 μmol/L	2.549 (0.994,6.536)	0.051		
Creatinine > 177 μmol/L	1.297 (0.461,3.652)	0.623		
Continuous renal replacement therapy	10.431 (3.334,32.635)	0.000*	5.755 (1.691,19.589)	0.005*
Mechanical ventilation	3.475 (1.801,6.706)	0.000*	1.411 (0.689,2.89)	0.347
Septic shock	6.376 (3.319,12.249)	0.000*	4.308 (2.085,8.901)	0.000*

HR, hazard ratio; CI, confidence interval. *P values are statistically significant.

**Figure 2:**
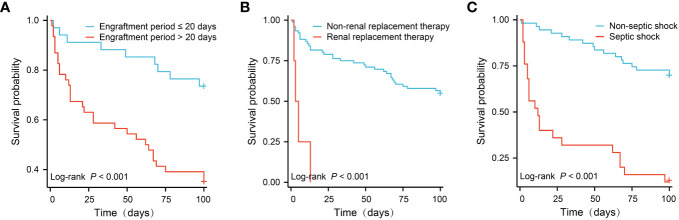
Survival comparison of patients with independent risk factors (Kaplan-Meier curve). **(A)** Engraftment period ≤ 20 days *vs.* > 180 days (65.2% *vs.* 26.5%, *P* < 0.001); **(B)** Non-renal replacement therapy *vs.* renal replacement therapy (100% *vs.* 46.1%, *P* < 0.001); **(C)** Non-septic shock *vs.* septic shock (88.0% *vs.* 30.9%, *P* < 0.001).

**Figure 2** showed the survival curves of independent risk factors. Patients with engraftment period > 20 days (26.5% *vs.* 65.2%, *P* < 0.001), CRRT (46.1% *vs.* 100%, *P* < 0.001) and septic shock (30.9% *vs.* 88.0%, *P* < 0.001) had significantly lower survival rates.

### 3.5 Construction of OS prediction model for HSCT recipients with MBI

**Figure 3:**
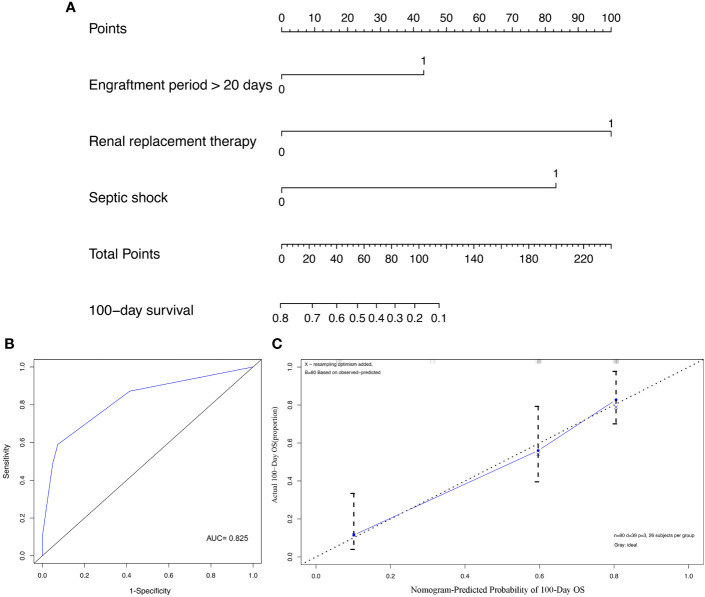
Construction and validation of prediction model for overall survival (OS). **(A)** The nomogram of OS; **(B)** The receiver operating characteristics (ROC) curve of nomogram; **(C)** The calibration curve of nomogram.

In order to quantitatively predict the 100-day survival rate of HSCT recipients with MBI, we established a nomogram (**Figure 3A**) using the independent risk factors related to death screened above, together with gender and age. The C-index and AUC of this model was 0.777 and 0.825, respectively (**Figure 3B**). The calibration curve indicated that there was a good consistency between actual observations and model predictions (**Figure 3C**).

## 4 Discussion

HSCT recipients are at high risk of infection (Styczynski et al., 2020). MBI often leads to poor prognosis due to the variety of strains involved and the complex mechanisms of drug resistance. Our results indicated that compared with single bacterial infection, HSCT recipients with MBI had a longer platelet engraftment time and a higher risk of adverse outcomes such as sepsis shock and death. The time interval from diagnosis to transplantation > 180 days and ICU admission after transplantation were independent risk factors for MBI acquisition.

In previous reports, we have repeatedly emphasized the disadvantages of prolonged transplantation interval: disease recurrence, repeated chemotherapy, bone marrow suppression, immunosuppression, mucosal damage, impaired organ function, excessive psychological pressure, delayed donor cell implantation, etc. (Liu et al., 2022; Jia et al., 2023). Kikuchi M et al. also confirmed that the interval between diagnosis and transplantation was a risk factor for BSI in the pre-engraftment period of HSCT patients (Kikuchi et al., 2015). That’s why the guidelines recommend early HSCT for patients with hematological malignancies who meet physical conditions (Blackburn et al., 2019). However, limited by age, financial burden, family support, medical resources/level and other factors, there are still a lot of patients who cannot receive transplantation at the most appropriate time. In particular, as the birth rate declines and the population ages, the number of HLA-matched sibling donors is decreasing. Fortunately, with the improvement of haplotype HSCT technology in recent years, the problem of donor source has been greatly solved, resulting in a rapid increase in the number of HSCT cases (Xu et al., 2018).

Undoubtedly, ICU gathered severe patients from different departments in the whole hospital, including trauma, post-surgery, multiple organ failure, etc., so the source of pathogens is very extensive. In addition, invasive procedures such as intravenous catheterization, urethral catheterization, tracheal intubation, incision and drainage further increase the risk of MBI. Reports indicate that 5%-55% of patients will be admitted to ICU after HSCT (Afessa and Azoulay, 2010). Sepsis/septic shock, acute respiratory failure, acute renal failure, mind change, GVHD and veno-occlusive disease are the most common causes. Studies from Barlas T et al. also showed that the incidence of BSI/catheter-related BSI in HSCT patients admitted to ICU was significantly increased (Barlas et al., 2021).

Engraftment period > 20 days, CRRT and septic shock were 3 independent risk factors for death in patients with MBI. Kikuchi M et al. reported that the cumulative incidences of pre- and post- engraftment BSI were 38.9% and 17.2%, respectively. Pre-engraftment BSI was associated with an increased risk of death. The OS at day 180 for patients with or without pre-engraftment BSI was 70.0% and 82.7% (Kikuchi et al., 2015). It proves that delayed engraftment will increases the probability of infection-related death in transplant patients. Not only that, but the extension of engraftment time means patients need to experience a longer period of agranulocytosis. During this period, their immunity was extremely low, unable to resist the invasion of various pathogens, and some opportunistic infections also break out at this time (Omrani and Almaghrabi, 2017; Suwabe et al., 2022). Moreover, infection may induce or aggravate GVHD, leading to transplant failure and even patient death (Kolb et al., 2018; Mori et al., 2018).

CRRT is a continuous, slow, isotonic extracorporeal circulation technique which can help the body remove excess water and certain solutes (endotoxin, inflammatory cytokines, middle molecular substances, etc.) (Tandukar and Palevsky, 2019). It is an important part of blood purification technology. After 2000, the clinical application of CRRT was no longer limited to renal replacement, but expanded to multiple organ support therapy (Ricci et al., 2018). However, a series of complications may occur during CRRT, such as bleeding, thrombosis, infection, nutritional loss, hypotension, hypothermia, electrolyte and acid-base imbalance (Elbahlawan et al., 2021; Kovvuru and Velez, 2021). Especially in the vulnerable population after transplantation, these risks are further amplified. For example, low platelet counts will increase the probability of bleeding or even lead to hematoma formation during subcutaneous puncture and Seldinger catheterization. Vascular connection device, rehydration connection device, extracorporeal circulation and prolonged indwelling of venous catheters also greatly increased the risk of infection. Studies have shown that pipeline joints and exposed parts of pipelines are the most common invasion sites of bacteria (Zhang et al., 2016; Fisher and Mokrzycki, 2023). In addition, excessive ultrafiltration volume and replacement fluid composition deviation can also affect the patient’s vital signs, dealing a fatal blow to the already serious illness. Neofytos D et al. found in a multicenter prospective analysis that hemodialysis was associated with reduced survival due to infection in adult HSCT recipients (Neofytos et al., 2009). Elbahlawan L et al. also found that the mortality rate of children undergoing CRRT after HSCT was as high as 52%-65% (Elbahlawan et al., 2021). It suggests that doctors should strictly control the indications of CRRT and closely monitor the possible complications in the process to ensure patient safety.

Sepsis shock is a systemic infection with acute onset and critical condition (Cecconi et al., 2018). Because of a large amount of inflammatory cytokines entering the blood and insufficient tissue perfusion, resulting in severe circulatory disorders and abnormal cell metabolism. The patient presents with persistent hypotension and hyperlactatemia that are difficult to correct. As the most serious complication of infection, septic shock has become one of the major global healthcare problems, affecting millions of people each year and causing one-third to one-sixth of deaths (Evans et al., 2021). Our data showed that the incidence of septic shock in HSCT recipients with MBI was 31.2%. Since the current research on post-transplantation infection is mostly focused on a single strain or a certain category, such as GNB or GPB, the available literature on MBI is limited, and we cannot fully compare this data with other transplantation centers for the time being. A recent report by Facchin G et al. pointed out that although the incidence of septic shock caused by polymicrobial bloodstream infections in HSCT patients was only 8%, the associated mortality rate was as high as 75% (Facchin et al., 2022).

In this study, CoNS + *E. coli*/*A. baumannii*/*P. aeruginosa* and *K. pneumoniae* + *A. baumannii* were the main combination of mixed infections. Consistent with previous reports, CoNS and *E. coli* were the most frequent isolates of GPB and GNB, respectively (Macesic et al., 2014; Forcina et al., 2017). What is different is that Trifilio S et al. found that the combination of clostridium difficile + gram-positive/negative bacteria was the most common pattern of polymicrobial bacterial infection in HSCT patients, and was thought to be associated with an increased risk of acute gastrointestinal graft-versus-host disease (Trifilio et al., 2015). Sy A et al. showed that Staphylococcus species (22.0%), Pseudomonas (12.1%) and Escherichia Coli (8.9%) were the main components of late polymicrobial bacterial infection in transplant recipients (Sy et al., 2021). We speculate that this may be related to a variety of factors such as primary disease, pre-treatment regimen/intensity, antibiotic prevention, HSCT type and so on. Besides, the gradual transformation of major pathogenic bacteria worldwide may also contribute to this difference (Sahitya et al., 2021).

CoNS is derived from the inherent symbiotic flora of skin and gut, and can be migrated or disseminated through exogenous channels such as catheters (Argemi et al., 2019). Our antibiotic susceptibility tests showed that CoNS was completely sensitive to vancomycin and linezolid, and slightly resistant to penicillin and aminoglycosides. This was somewhat different from what Niyazi D previously reported (Niyazi et al., 2022): They found CoNS resistance rates to penicillin, erythromycin, gentamicin, and ciprofloxacin all exceeding 60%, significantly higher than our statistical results. We speculate that this may be caused by the high proportion of methicillin-resistant CoNS in their study cohort. In contrast, the drug resistance of GNB was much more severe: only tigecycline and amikacin maintained relatively high sensitivity as a whole, but the anti-infection effect of the former against *P. aeruginosa* and the latter against *S. maltophilia* was also very poor. The resistance rates of *P. aeruginosa* and *A. baumannii* to carbapenems were more than 58% and 85%, respectively, higher than 37.9% and 63.6% reported by Averbuch D (Averbuch et al., 2017). It may be related to the type of primary disease, inappropriate empirical antimicrobial therapy and more multidrug-resistant strains. The above data show that GNB has a greater impact on MBI. Fortunately, in recent years, some new antibiotics, such as Ceftazidime avibactam, Ceftolozane/Tazobactam, Meropenem-vaborbactam, Imipenem/Cilastatin/Relebactam, etc., have been proved to have good efficacy and high safety against GNB, especially carbapenem resistant GNB (Pogue et al., 2020; Alosaimy et al., 2021; Clerici et al., 2021; Heo, 2021). Unfortunately, due to the large time span of this study, many antimicrobial susceptibility tests could not be performed at that time. Additionally, factors such as time to market, drug prices, and patients’ economic affordability limited the use of these novel antibiotics, which increased the difficulty of collecting drug sensitivity results, and ultimately made these data unable to be included in statistical analysis. Different from previous reports that the degree of HLA matching, aGVHD, mechanical ventilation and MDR increase the infection-related mortality of HSCT recipients, here, we did not find statistical differences in the above factors between the survival group and the death group (Forcina et al., 2017; Chueansuwan et al., 2018; Liu et al., 2018; Styczynski et al., 2020).

Admittedly, our study had some limitations. First, it is a single-center retrospective design, and the results may not be well generalized to other transplant centers due to population heterogeneity. Secondly, the number of patients included in this study was relatively small. Thirdly, all patients received antibiotic prophylaxis, but this practice was not completely uniform across the transplant center. Finally, although the prognostic model was adjusted for many key clinical covariates, we cannot exclude the influence of other unmeasured or temporarily unknown factors.

## 5 Conclusion

In summary, HSCT recipients with MBI had severe antibiotic resistance and high mortality. Engraftment period > 20 days, CRRT and septic shock were independent risk factors associated with mortality. Therefore, shortening transplant waiting time, promoting donor stem cell implantation as soon as possible, strictly controlling CRRT indications, and early identification and timely correction of shock may help improve the prognosis of HSCT patients.

## Data availability statement

The original contributions presented in the study are included in the article/supplementary material. Further inquiries can be directed to the corresponding author.

## Ethics statement

The studies involving human participants were reviewed and approved by the Institutional Review Committee of Xiangya Hospital. Written informed consent for participation was not required for this study in accordance with the national legislation and the institutional requirements.

## Author contributions

YJ and YFL were involved in the study conception and design. YL, YFL, and XFC participated in the data management. All authors participated in the interpretation and statistical analysis of data. YFL wrote the draft and YJ revised it. All authors contributed to the article and approved the submitted version.
